# Self-Assembly of RGD-Functionalized Recombinant Spider Silk Protein into Microspheres in Physiological Buffer and in the Presence of Hyaluronic Acid

**DOI:** 10.1021/acsabm.3c00373

**Published:** 2023-08-14

**Authors:** Eirini Ornithopoulou, Carolina Åstrand, Linnea Gustafsson, Thomas Crouzier, My Hedhammar

**Affiliations:** † Department of Protein Science, School of Chemistry, Biotechnology and Health (CBH), KTH Royal Institute of Technology, SE-106 91 Stockholm, Sweden; ‡ Spiber Technologies AB, Roslagstullsbacken 15, 114 21 Stockholm, Sweden; § Division of Micro and Nanosystems, School of Electrical Engineering and Computer Science (EECS), KTH Royal Institute of Technology, SE-106 91 Stockholm, Sweden; ∥ Department of Chemistry, School of Chemistry, Biotechnology and Health (CBH), KTH Royal Institute of Technology, SE-106 91 Stockholm, Sweden

**Keywords:** recombinant spider silk, self-assembly, silk microspheres, hyaluronic acid, confocal microscopy, fluorescence microscopy, cryo-electron microscopy, cell culture

## Abstract

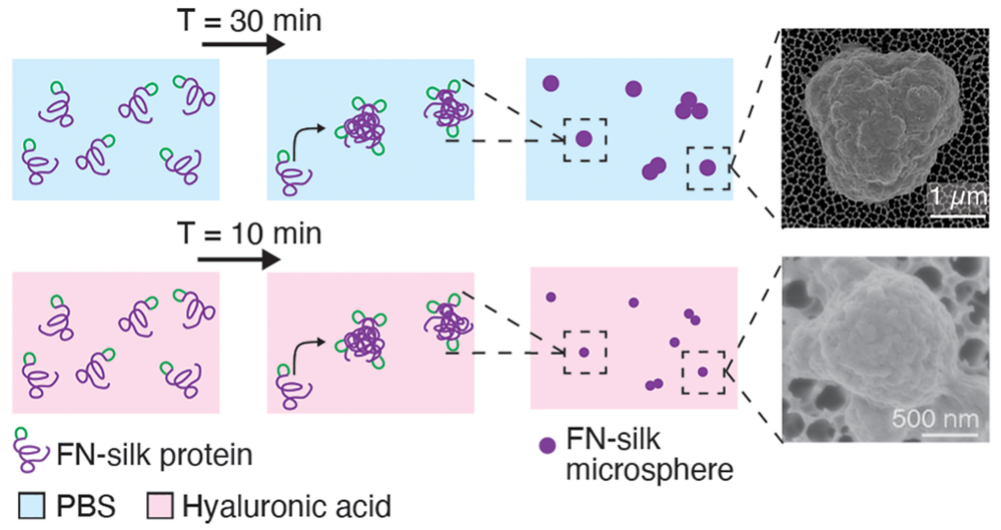

Biomaterials made of self-assembling protein building blocks are widely explored for biomedical applications, for example, as drug carriers, tissue engineering scaffolds, and functionalized coatings. It has previously been shown that a recombinant spider silk protein functionalized with a cell binding motif from fibronectin, FN-4RepCT (FN-silk), self-assembles into fibrillar structures at interfaces, i.e., membranes, fibers, or foams at liquid/air interfaces, and fibrillar coatings at liquid/solid interfaces. Recently, we observed that FN-silk also assembles into microspheres in the bulk of a physiological buffer (PBS) solution. Herein, we investigate the self-assembly process of FN-silk into microspheres in the bulk and how its progression is affected by the presence of hyaluronic acid (HA), both in solution and in a cross-linked HA hydrogel. Moreover, we characterize the size, morphology, mesostructure, and protein secondary structure of the FN-silk microspheres prepared in PBS and HA. Finally, we examine how the FN-silk microspheres can be used to mediate cell adhesion and spreading of human mesenchymal stem cells (hMSCs) during cell culture. These investigations contribute to our fundamental understanding of the self-assembly of silk protein into materials and demonstrate the use of silk microspheres as additives for cell culture applications.

## Introduction

Protein based materials are multipotent and highly versatile as they can be used in a wide range of applications such as drug delivery,^1^ tissue engineering,^2^ depollution materials,^3^ bioelectronics,^4^ textiles,^5^ and food science.^6^ Natural spider silk is one of nature’s superior protein materials due to its physical properties of high tensile strength while maintaining extensibility.^7,8^ Spider silk has been explored as a wound suture material^9^ and support for nerve regeneration,^10^ showing both biocompatibility and stability. Taken together, spider silk is a great candidate for many biomedical applications.

There are, however, unsurpassed thresholds in mass-producing natural spider silk since it is challenging to farm and harvest from spiders,^11^ which is why recombinant DNA technology has been employed to develop artificial spider silk materials from key parts of the sequence of various silk proteins (spidroins).^12,13^ Silk-like materials based on recombinant silk proteins successfully deliver the advantages of the natural material with additional properties, such as scalability,^14,15^ biofunctionalization,^16−19^ tunability,^20,21^ low immune response, and improved biocompatibility.^22,23^

Recombinant gene fusion technology can further be used to functionalize the silk-like materials by adding short motifs or protein domains covalently linked to the expressed protein.^24^ Functionalization with cell binding motifs derived from natural extracellular matrix (ECM) proteins,^25−27^ such as RGD^26^ and IKVAV,^28^ have been demonstrated to increase cell adhesion and proliferation capabilities of seeded cells.^29−31^

In this study, we use the functionalized recombinant protein FN-4RepCT,^19^ herein denoted FN-silk, consisting of four repeats of the repetitive sequence and the C-terminal domain of the major ampullate spidroin 1 (MaSp1) from *Euprosthenops australis*,^32^ combined with an RGD-motif on a turn loop as it is naturally presented in fibronectin (FN). FN-silk has previously been shown to form fibrillar coatings on liquid–solid interfaces^33^ and membranes at the air–liquid interface.^34^ Moreover, the assembly at air–liquid interfaces has been utilized to form fibers,^35^ foams,^36^ and nanowires.^37^ Herein, we investigate the self-assembly of FN-silk into microspheres, which occurs within the medium instead of at interfaces.

Microspheres have previously been prepared from other recombinant silk proteins derived from their respective spidroins, e.g., major ampullate dragline spidroin from *Araneus diadematus* (ADF4),^38^ tubuliform spidroin 1 (TuSp1) from the black widow spider,^39^ and major ampullate spidroin 1 (MaSp1)^40^ and 2 (MaSp2) from *Nephila clavipes*.^41,42^ In the case of eTuSp1 from TuSp1, a HFIP-on-oil method followed by ethanol treatment and evaporation was used to produce spheres,^39^ similarly to how silk capsules have previously been produced.^43^ However, the most common method of inducing microsphere formation using spidroins has been *salting-out* which consists of introducing high concentrations of potassium phosphate (up to 2 M),^44^ with varying pH of the potassium phosphate buffer (pH 4–10).^41^ It has been proven possible to acquire silk microspheres with combined properties by blending two different recombinant constructs (from MaSp1 and MaSp2).^45^ Interestingly, in one of the studies, using MS2(9x) from MaSp2, it was shown that the purification method, thermal or acidic extraction, affected the size and other features of the spheres obtained by the following salting-out procedure.^42^ In the aforementioned cases using salting-out methods, the obtained microspheres have been shown to be solid with a size distribution of 0.4–3.0 μm, to have a high β-sheet content (>60%), and to be colloidally stable in solution. There have also been indications of porosity within the microspheres,^42,45^ potentially due to an underlying fibrillar framework.

Herein, we investigate how the FN-silk protein spontaneously forms micrometer-sized spheres in the bulk of a physiological buffer (1× PBS, i.e., 10 mM Na_2_HPO_4_, 1.8 mM KH_2_PO_4_, 137 mM NaCl, and 2.7 mM KCl). Moreover, we investigate how the addition of the extracellular matrix glycosaminoglycan component hyaluronic acid^46^ affects the assembly of FN-silk, both in gel solution and when cross-linked into a self-standing gel.

In order to observe the self-assembly of the FN-silk microspheres in different environments, we used optical and fluorescence microscopy. Thereafter, we isolated the obtained microspheres using centrifugation and following washes. To characterize the silk microspheres morphologically and assess their size distribution, we used confocal microscopy, as well as electron microscopy. For the intrinsic mesostructure of the microspheres, we gained insights by milling with a focused ion beam (FIB) in cryogenic conditions and imaging with scanning electron microscopy (SEM), and by performing cryo-ultramicrotomy and transmission electron microscopy (TEM) on 80 nm sections of the spheres. In order to characterize the secondary structure, we employed circular dichroism (CD) spectroscopy, attenuated total reflection-Fourier transform infrared (ATR-FTIR) spectroscopy, and tracking of the self-assembly with a luminescent conjugated oligothiophene stain (Amytracker). Finally, we explored the use of FN-silk microspheres for *in vitro* culture of human mesenchymal stem cells (hMSCs) in 2D and 3D (cell spheroids) cultures.

## Materials and Methods

### Preparation and Isolation of Silk Microspheres

Recombinant silk protein FN-4RepCT (FN-silk) (with a purity >90%, determined with Bioanalyzer) was obtained from Spiber Technologies AB at 3.2–3.3 mg/mL in PBS. The protein solution was stored at −80 °C and thawed in a sealed tube at room temperature prior to use. For fluorescence microscopy analyses, 10% fluorophore labeled (see below) silk protein was added by gentle pipetting. Directly after thawing, the protein was diluted to 1 mg/mL in 1× PBS buffer, or 1.6% HA, depending on the intended experiment. The sample was left undisturbed for 30–60 min in a sealed Eppendorf tube. For isolation of microspheres, the solution was transferred to a new tube to remove any membrane and coating formed at the interfaces during the incubation period. The solution was then centrifuged, using a VWR Microstar17 tabletop centrifuge, at room temperature for 15 min at 16200*g*. The supernatant was aspirated gently, and the pellet was resuspended in PBS corresponding to the initial volume. A second centrifugation was performed at 16200*g* for 15 min. The supernatant was gently removed, and the pellet resuspended in water. The isolated silk microspheres were stored at 4 °C until use.

### Fluorophore Coupling to FN-4RepCT

In order to covalently couple a fluorophore to the N-terminus of FN-silk via amine chemistry, DyLight 488 NHS Ester (Thermofisher) was used. The dye was thoroughly dissolved in DMF to 10 mg/mL and mixed gently with the silk solution at 4 °C, in a molar ratio of 1:1. After 30 min, desalting of the sample was performed using a PD-10 column (Cytiva), according to the supplied gravity flow protocol. The flow through was collected, sterile filtered, and aliquoted in a laminar flow hood and then stored at −80 °C.

### Modifications of Hyaluronic Acid

Hydrazide-modified hyaluronic acid (HA-CDH) was prepared essentially according to a previously published protocol.^47^ Briefly, 1 mmol of HA (150 kDa Lifecore Biomedical, LLC, Minnesota, USA) was mixed with 1 mmol of hydroxybenzotriazole (HOBt), in 100 mL of water in ambient conditions and under stirring. After 2 h, 1 mmol of carbodihydrazide (CDH) was added, and the pH was adjusted to 4.7–4.9, followed by the addition of 0.25 mmol of 1-ethyl-3-(3-(dimethylamino)propyl) carbodiimide (EDC). After dissolution, the solution was loaded into a dialysis bag (Spectra Por-6, MWCO 3500) and dialyzed against HCl 0.01 M (pH 3) containing 0.1 M NaCl (2 × 2 L, 48 h), then dialyzed against deionized water (24 h). Finally, the solution was lyophilized, and HA-CDH was obtained. The HA-CDH modification was confirmed with a TNBS assay, using *tert*-butyl carbazate for the standard curve, and was estimated to be approximately 20–25% (1 of every 4–5 dimeric HA moieties were modified). Aldehyde-modified hyaluronic acid was prepared essentially as in the previously published protocol.^48^ This component, mixed with the HA-CDH, forms hydrazone linkages (cross-links) that result in a self-standing gel in less than a minute. The modification was achieved as follows: HA was dissolved with HOBt, as in the CDH protocol. Then, 2 mmol of 3-amino-1,2-propanediol was added to the solution, and the pH was adjusted to 6.0 using HCl. Next, 0.5 mmol of EDC was added, which theoretically corresponds to 21% modification.^48^ The mixture was stirred overnight. The solution was dialyzed similar to the CDH modification for 48 h, and then with HCl 0.01 M (pH 3) for 24 h and then against water for 24 h. After that, 1 mmol of NaIO_4_ dissolved in deionized water was added dropwise under stirring and left for 10–15 min. The excess NaIO_4_ was quenched by 1.4 mL of ethylene glycol under stirring for 2 h. Then the solution was dialyzed against water for 48 h with several changes, and then the solution was lyophilized.

### Optical and Fluorescence Microscopy and Time Lapse Observations

An inverted Leica DMI6000 B microscope was used in two modes: fluorescence with the L5 filter and bright field. For time lapse experiments, image capture was set to occur automatically every 30 s over at least the first hour of incubation and self-assembly, to observe appearance and motion of the spherical particles in the wet native state of each condition. Three droplets (20 μL) of FN silk (1 mg/mL) were incubated inside the same glass-bottomed Petri dish, one in PBS, one in HA-CDH, and one in HA-gel.

### Confocal Microscopy

Confocal microscopy was used to measure the size distribution of the microspheres in their native wet state. For confocal microscopy, a Zeiss LSM980-Airy2 inverted microscope was used with an immersive oil lens, 63×. Image collection was done in the form of *z*-stacks to determine the sphere size and dispersity. Slice intervals were set to 0.23–0.25 μm. Master gain was approximately 500 V, and 488 laser intensity was 0.6–1.1%, depending on eliminating the saturation of pixels. The pinhole was approximately 1 Airy Unit. For analyzing the data and determining the size distribution of the silk microspheres, the Fiji suite for ImageJ was used, using the Measure feature. In the case of measuring microsphere size inside the cross-linked HA gel, the maximum projection of each pixel was used to produce one slice with all the spheres, and the find edges function was used. For each condition, 150 measurements of the microsphere diameter were taken from 2 or 3 experiments in each case.

### Cryogenic Focused Ion Beam Scanning Electron Microscopy (Cryo-FIB SEM)

Cryo-FIB SEM was used for imaging and sectioning of the microspheres. A droplet (20 μL) of microspheres, isolated as described above, was air-dried onto an aluminum filter with a 0.2 μm mesh size (Merck) and then washed with deionized water to rinse away residuals of salts or HA. The instrument used was Thermofisher Scientific Scios cryo-FIB, at the Centre for Electron Microscopy at Umeå University. Cross sections were made at 30 kV Ga^+^ ions and 10 pA current. Prior to being milled, the dried samples were sputter-coated with a 10 nm layer of Pt.

### Transmission Electron Microscopy (TEM) and Cryo-ultramicrotomy

Small samples (1 μL) of microspheres isolated according to the protocol were placed on the pin holder and plunge frozen (vitrified) in liquid nitrogen. Sections of 80 nm thickness were prepared at −120 °C using a Leica EM UCF7 microtome equipped with a cryochamber. The sections were transferred onto hexagonal copper grids coated with Formvar and carbon. After that the grids were stained with uranyl acetate and air-dried. Imaging was carried out at room temperature using a TEM-Talos (FEI).

### Circular Dichroism

The instrument model used in this work was qCD Chirascan by Applied Photophysics. For the measurements and data collection, Chirascan software was used, and for the analysis and visualization, Microsoft Excel was used. The measurements were carried out under ambient conditions. The spectra were collected in the range of 190 to 260 nm with a 1 nm step and a 1 s duration for each measurement, repeated thrice, and averaged. A background for the buffer was measured but ignored, as it was very close to zero everywhere in the regions of interest. At regions close to 190–205 nm, there was some noise attributed to the glass cuvette and buffer which was thus excluded. For CD spectroscopy, samples with 0.8% m/V HA were used.

### Attenuated Total Reflection-Fourier Transform Infrared (ATR-FTIR) Measurements

An ATR-FTIR Vertex 70 FTIR Spectrometer (Bruker) equipped with a HgCdTe detector was used and continuously purged with dry air to prevent water vapor interference. A Diamond crystal Bruker Platinum ATR unit was pressed onto the crystal with a piston. The OPUS software was used for collection. Between each sample, the background signal was measured and subtracted. For sample preparation, microspheres from PBS, isolated according to the protocol, were laid onto a borosilicate glass slide in 7 layers of 5–10 μL solution, deposited consecutively, and left to dry. Two replicates were prepared and measured, the background was removed, and the results were averaged.

### Luminescent Conjugated Pentameric Formyl Thiophene Acetic Acid (LCO) Staining (Amytracker 680)

An LCO stain for β-sheet structures, commercially available as Amytracker 680, purchased from Ebba Biotech AB, was used to visualize the self-assembly of the soluble FN-silk protein into microspheres. The preparation of the FN-silk solution followed the previous protocols for self-assembly (1 mg/mL of freshly thawed silk in PBS), and the dye was added to the solution from the beginning at a 1:1000 dilution. Fluorescence microscopy imaging was initiated at regular intervals of 5 min, up to 45 min. A control with just Amytracker 680 and PBS showed no fluorescent particles (data not shown).

### Culture of hMSCs with Microspheres in 2D and 3D Cultures

Human bone marrow-derived mesenchymal stem cells (hMSCs; ScienCell) were cultured in T75 flasks (Corning) precoated with BioLaminin 521 (BioLamina) using mesenchymal stem cell growth medium (PromoCell) for expansion. The medium was changed every 2–3 days, and the cells were passaged using TryplE express enzyme (Gibco) when they reached around 75–85% confluency. Passages 3–6 were used for the experiments described below.

For culture in 2D, 5 μL droplets with 22 μg of microspheres from labeled FN-silk were added onto each well of a hydrophobic 24-well plate for suspension culture (Sarstedt) and allowed to dry. The surfaces were rinsed once with PBS before seeding of 25 000 cells/cm^2^. As a negative control, wells without microspheres were used.

For culture in 3D, 7000 hMSCs were added as a single cell suspension into a U-bottom Ultra low attachment 96-well plate (Nunclon Sphera) with 10 μg of microspheres from labeled FN-silk in 150 μL of growth medium. In the control, the same number of cells were added in 150 μL of growth medium without microspheres. After 1 day, the cell spheroids were transferred into a 48-well plate with fresh medium and cultured individually on a shaking stand at 95 rpm for 5 days. The medium was changed every second day. Four hMSC spheroids per condition were harvested at days 1 and 5 after cell seeding.

### Immunocytochemistry Analyses of hMSCs and FN-silk

Cells or cell spheroids were washed once in PBS and fixed using 4% paraformaldehyde at RT for 10 min (cells grown on 2D surface) and 15 min (cell spheroids). This was followed by three washes in PBS. Permeabilization was done for 15 min using PBS with 0.1% Triton X-100 followed by blocking for 1 h with 10% donkey serum (Jackson ImmunoResearch) in PBS with 0.1% Tween-20 (PBST). The samples were incubated with Phalloidin-Alexa 647 (8940S, Cell signaling, 1:80) for 1 h at RT before three washes in PBST. Nuclei were visualized with DAPI (Sigma). Imaging was performed on a fluorescent microscope, Leica DMI6000B. Images were processed using NIH ImageJ software.

## Results and Discussion

### Time Course of Self-Assembly of FN-silk into Microspheres

**Figure 1:**
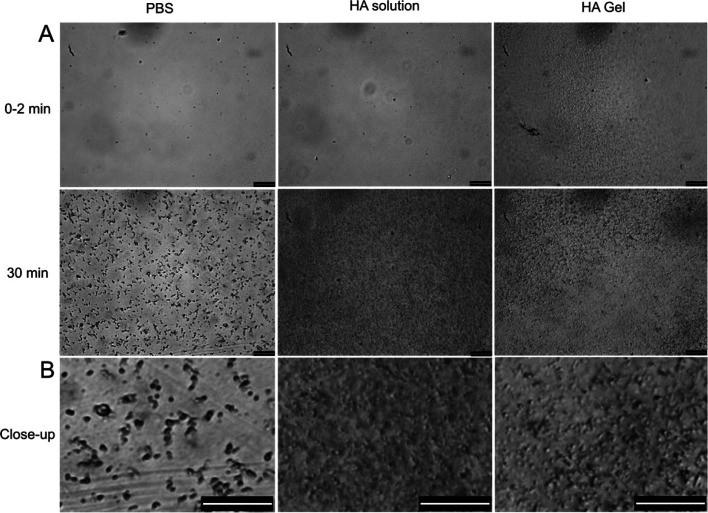
Optical imaging of self-assembly of FN-silk into microspheres. (A) Top row: Images taken directly (0–2 min) after sample preparation of FN-silk (1 mg/mL) in 1× PBS buffer (PBS), hyaluronic acid (1.6%) dissolved in PBS (HA-solution), and HA cross-linked into a gel (1.6% HA-gel). Lower row: Images of the respective conditions after 30 min of incubation. (B) Digitally magnified parts of the bright field images of each of the three conditions after 30 min. Scale bar 30 μm.

The FN-silk protein has previously been utilized to form fibrillar membranes at liquid/air interfaces, with varying thickness depending on the protein concentration used.^49^ We recently observed that during overnight incubation used for membrane formation, some of the FN-silk protein assembled into microspheres in the bulk. More microspheres were found from solutions with a higher concentration of FN-silk (SI Figure S1). In order to observe the process of self-assembly into microspheres over time, FN-silk protein was diluted to 1 mg/mL either in just 1× PBS buffer (PBS) or PBS supplemented with 1.6% hyaluronic acid (HA). Two chemically modified HA components were made and used in two different conditions, either the HA-CDH component alone, which remains a solution (HA-solution), or both HA-CDH and HA-ALD, which cross-link into a hydrogel (HA-gel). The two components carry an aldehyde group and a hydrazide group, respectively, that spontaneous react and within seconds result in a self-standing gel (see Materials and Methods for more information). Droplets of FN-silk mixed with the three different components (PBS, HA-solution, and HA-gel) were prepared and placed in a saturated environment to prevent evaporation. Optical microscopy imaging was performed at 30 s intervals, in both differential interference contrast (DIC) and fluorescence mode, to look for the first signs of formation, clustering, and following sedimentation of the silk microspheres (Figure 1, Table 1, SI Figure S2). It should be noted that according to the Abbe diffraction limit, coined in 1873,^50^ objects below 200 nm would not be detected with this method due to physical limitations, which is why events below that resolution, e.g. during a possible nucleation phase, are undetectable. The observations made here are of micrometer sized objects and serve to compare the FN-silk’s behavior in the three conditions tested.

**Table 1:** Observations of Microsphere Population at Different Time Points for the Three Different Conditions^a^

time (min)	PBS	HA-solution	HA-GEL
0–2	none	many	many/saturated
5	none	many	saturated
10	some	many/saturated	saturated
20	some	many/saturated	saturated
30	many	many/saturated	saturated
40	many	many/saturated	saturated
45	many/saturated	many/saturated	saturated

a None = absence of microspheres, some = a small population (10-250), many = a large population (1000+), and saturated = the sample was full of microspheres with no observable increase beyond that time point.

In the case of FN-silk in PBS, a few microspheres started to appear after a couple of minutes, which then increased in number as time passed. After 20–30 min, these microspheres and clusters thereof sedimented and formed a layer at the bottom of the sample. When FN-silk protein was added to a HA solution instead, microspheres appeared almost immediately within the first minutes. The microspheres remained more spread out throughout the bulk due to the increased viscosity of the medium. However, at later time points, after 10–20 min, sedimentation of microspheres at the bottom of the sample could be observed. When FN-silk was added during the formation of a HA-gel via cross-linking, microspheres appeared immediately. The microspheres were seen moving somewhat inside the medium until after a few minutes when the gelation was complete and visible movement was halted. After this time point, no sedimentation and no further coalescence or clustering of the microspheres could be observed. The microspheres remained equally spread out within the medium, at least for 6 days of storage (SI Figure S3).

Overall, the results showed that FN-silk formed microspheres much faster in the presence of HA, especially when it was inside a cross-linked HA gel. This phenomenon could be attributed to several factors. For example, the concentration of the FN-silk protein could be increased locally due to steric hindrance within the hydrogel, which increases the likelihood of protein–protein interactions and thus the rate of self-assembly. The HA molecules could further provide a scaffold for the protein molecules to organize and align upon, thereby promoting hydrophobic interactions between the nonpolar amino acids of the silk protein. Additionally, hyaluronic acid could provide charged or polar groups that form electrostatic interactions with parts of the FN-silk to increase the localization of the molecules further. To the best of our knowledge, a similar observation of the effects of HA on silk assembly has not been reported before.

### Isolation of FN-silk Microspheres Formed in PBS and HA Solution

In order to further characterize the FN-silk microspheres, a facile method for isolation was developed and validated. The protocol has not been optimized for maximal yield of microspheres but rather designed to be applicable for microspheres formed both in PBS and HA-solution. Microspheres formed in HA-gel were not possible to isolate in the same way since they were kinetically trapped inside the HA hydrogel.

**Figure 2:**
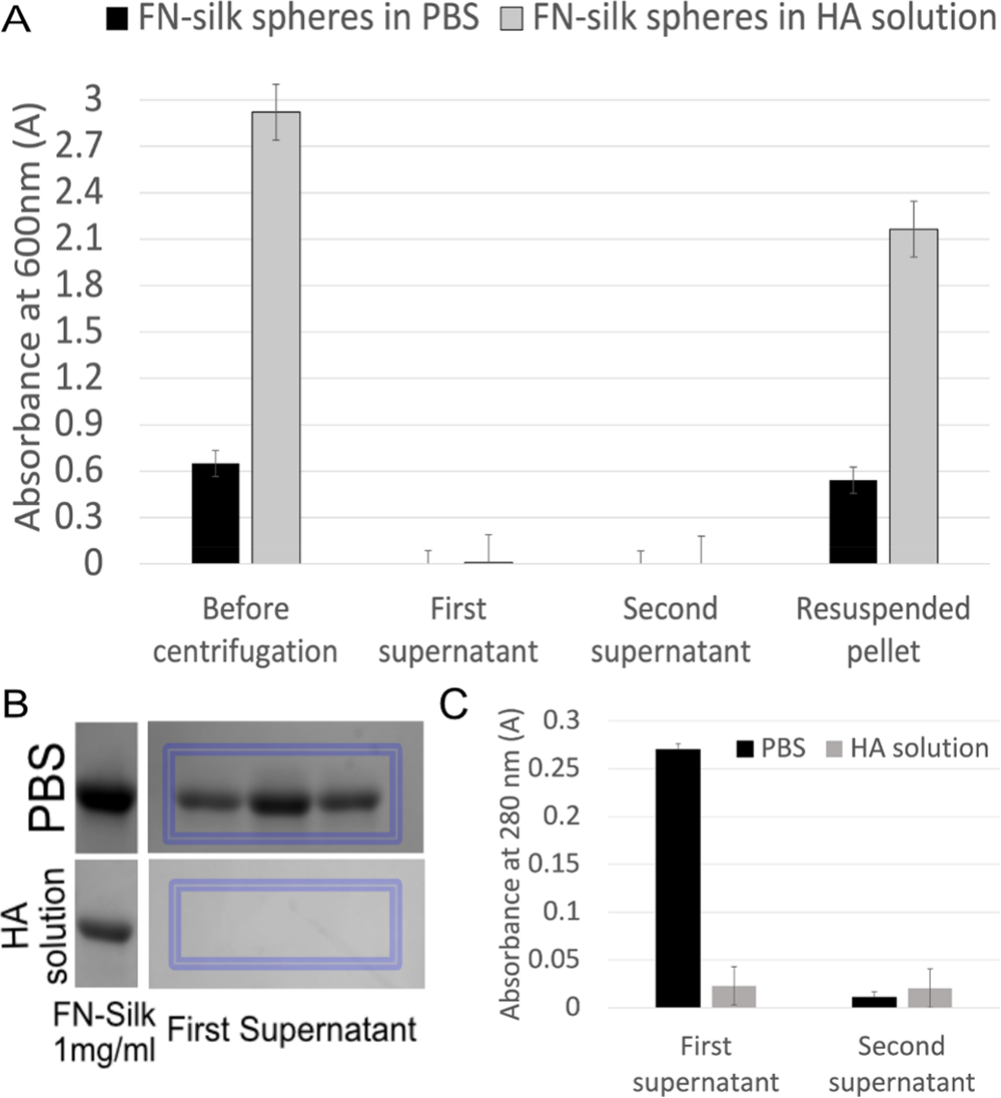
Evaluation of the process for isolation of the microspheres using centrifugation. (A) Turbidity (optical density at 600 nm) was used to compare the number of microspheres present in the solution at the different steps; directly after incubation (before centrifugation), supernatant after first centrifugation (first supernatant), supernatant after second centrifugation (second supernatant), and resuspension of the final pellet (resuspended pellet). (B) SDS-PAGE analysis of soluble FN-silk protein present in the supernatant after the first centrifugation. (C) Absorbance at 280 nm was used to determine the concentration of soluble FN-silk protein in the supernatants of PBS (black) and HA-solution (with background HA solution subtracted in the first supernatant) (gray).

From the previously described time course study, an incubation time of 30 min was chosen, so that microspheres were formed in both conditions. Measured turbidity (OD600) confirmed a larger fraction of microspheres in the HA-solution compared to in PBS (Figure 2A). Thereafter, the two solutions (PBS or HA) with microspheres were transferred to new tubes before being centrifuged at 16200*g* for two rounds followed by washes with PBS between rounds. The transfer aimed to eliminate the silk coating or particles attached to the walls of the first tube after the incubation. After the first centrifugation, the turbidity of the supernatant was almost zero in both conditions (Figure 2A), which means that the microspheres had successfully been sedimented into the pellet during centrifugation. Absorbance at 280 nm (Figure 2C) and SDS PAGE analysis (Figure 2B) revealed that the concentration of FN-silk protein in the first supernatant was estimated to be approximately 0.51 mg/mL in PBS and only 0.05 mg/mL in the HA solution, compared to the initial 1 mg/mL. The HA-solution gave a high background signal at 280 nm which has been subtracted when measuring the absorbance of the first supernatant (Figure 2C, gray). After the first centrifugation, the pellet was resuspended in PBS, and the microspheres were centrifuged once more at 16200*g* for 15 min. Again, turbidity very close to zero confirmed successful sedimentation. This second supernatant showed a low absorbance at 280 nm for both conditions (corresponding to 0.02 mg/mL soluble FN-silk in PBS), confirming the removal of the majority of soluble protein. The low and persistent signal from the supernatant from the HA-solution could be due to some remaining HA molecules, as SDS-PAGE showed no soluble silk protein even from the supernatant of the first round. The final pellet was then resuspended in Milli-Q water, and turbidity measurement (Figure 2A) showed retrieval of almost 83% and 74% of the microspheres formed during incubation in PBS and HA-solution, respectively.

### Size Distribution of the FN-silk Microspheres

**Figure 3:**
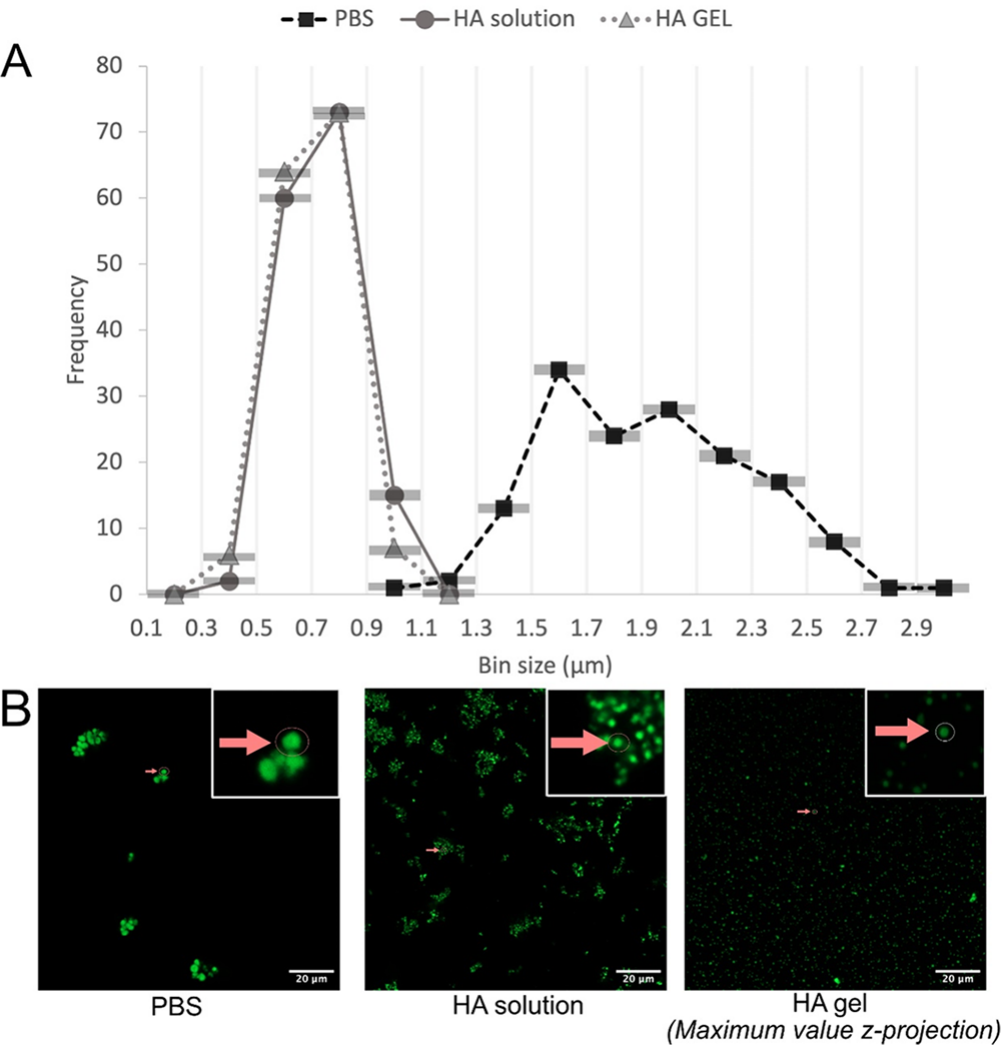
Confocal microscopy analysis of size distribution of the formed silk microspheres. (A) Histogram of the frequency of microspheres of each bin size, with intervals of 0.2 μm, self-assembled in the three different environments. Microspheres formed in PBS and HA-solution were isolated before imaging, while microspheres formed in cross-linked HA were imaged inside the gel. (B) Representative corresponding confocal images from the three conditions. Example of size measurements are shown with arrows in red. Micrograph of microspheres in HA-gel shows the maximum value *z*-projection of the stacks. Scale bar 20 μm.

Confocal microscopy analysis and ImageJ were used in order to measure the size and following statistical analysis for determining the distribution of the microspheres, using 0.2 μm bins (Figure 3). Microspheres formed in PBS and HA-solution were prepared according to the isolation protocol described above, while microspheres formed in the HA-gel were measured *in situ* within the gel. The imaging was done using confocal microscopy with the same laser wavelength (488 nm) as the fluorophore coupled to a fraction (10%) of the FN-silk protein. In order to ensure that the size measured is close to the actual size, *z*-stacks were captured with 0.23–0.25 μm intervals, and each sphere measured was in focus at its maximal diameter.

The obtained measurements showed an average size of single microspheres formed in PBS to be 1.84 ± 0.36 μm and only 0.61 ± 0.11 and 0.65 ± 0.12 μm for microspheres formed in HA-solution and HA-gel, respectively, only a third of the size of microspheres formed in PBS. Moreover, the size distribution of microspheres formed in PBS was approximately three times wider than that in the other two conditions. This could be attributed to more uncontrolled protein–protein interactions when microspheres were formed in just a buffer solution without any other biomolecules as well as a lower viscosity. Our results indicated that the addition of a long chain hydrophilic glycosaminoglycan, such as hyaluronic acid, can be used as a method of controlling the average size, size distribution, and dispersion of the self-assembled silk microspheres.

### Morphology and Mesostructure of the FN-silk Microspheres

**Figure 4:**
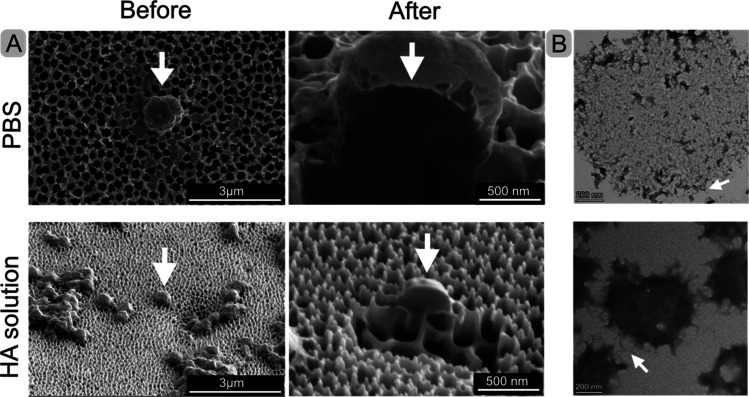
Electron microscopy analysis of the FN-silk microspheres. (A) Cryo-FIB SEM imaging of isolated FN-silk microspheres formed in PBS (upper row) and HA-solution (lower row). Single microspheres (arrow) before (left) and after (right) milling with FIB. (B) Cryo-TEM images of the FN-silk microspheres formed in PBS (upper) and HA-solution (lower) after cryo-ultramicrotomy. Arrows point to examples of fibrillar-like extensions from the silk microsphere.

Scanning electron microscopy (SEM) was used to get a better look at the surface morphology of the microspheres. Furthermore, focused-ion beam (FIB) milling was applied to look more closely at the mesostructure within the microspheres (Figure 4A). At first look, the surface roughness of the silk microspheres was more prominent in electron microscopy analysis, which offers significantly higher resolution (1000×) than light microscopy. However, as the herein used electron microscopy demands dry conditions and vacuum, it is important to be aware of possible artifacts. Upon milling individual spheres using FIB-SEM under cryogenic conditions, the inner part of the microspheres was revealed. The microspheres seemed homogeneously filled. This agrees with previous observations of recombinant spider silk spheres analyzed using FIB milling and SEM.^38,41,42^ Some indications of pores can be observed in the microspheres formed in PBS (Figure 4A, SI Figure S4). Similar mesostructure within the microspheres has been observed in other recombinant silk microspheres.^42,45^

Additionally, transmission electron microscopy (TEM) was used to look at 80 nm thick sections of the microspheres, obtained by performing cryo-ultramicrotomy of plunge-frozen microspheres followed by uranyl acetate staining. The resulting TEM images (Figure 4B, SI Figure S5) indicated a fibrillar and porous mesostructure, especially inside microspheres formed in PBS. Microspheres formed in HA were smaller and more compact with emanating fibril-like structures. These protrusions could consist mostly of FN-silk or could be caused by incorporation of HA molecules. These protrusions seem to be involved in the interconnection of the microspheres into clusters, indicated by the white arrows in Figure 4B. Additional TEM images of the sections in both conditions can be found in SI Figure S5.

Taken together, these results could be interpreted by a mechanism in which the self-assembling propensity of the amphiphilic FN-silk protein into microspheres in highly aqueous environments is initially driven by hydrophobic interactions. This might be followed by self-organization into more stable fibrillar β-sheet structures within the microspheres, similar to what has been observed recently in investigations of the FN-silk behavior at liquid/solid and liquid/air interfaces.^33,51^ A similar mechanism has been suggested before by Slotta et al.^38^ in the formation of microspheres from a recombinant spider silk protein by salting-out in high concentrations of potassium phosphate. They assert that in the initial stage of sphere formation, the liquid–liquid phase separation of aqueous and proteinaceous phases occurs and is maintained by the high ion concentration and that the secondary structure transition (β-sheet formation) and subsequent stabilization are similar in microspheres and in fibrils, based on the similarities in the secondary structure for the two formats.

### Protein Secondary Structure Transition during Formation of Silk Microspheres

**Figure 5:**
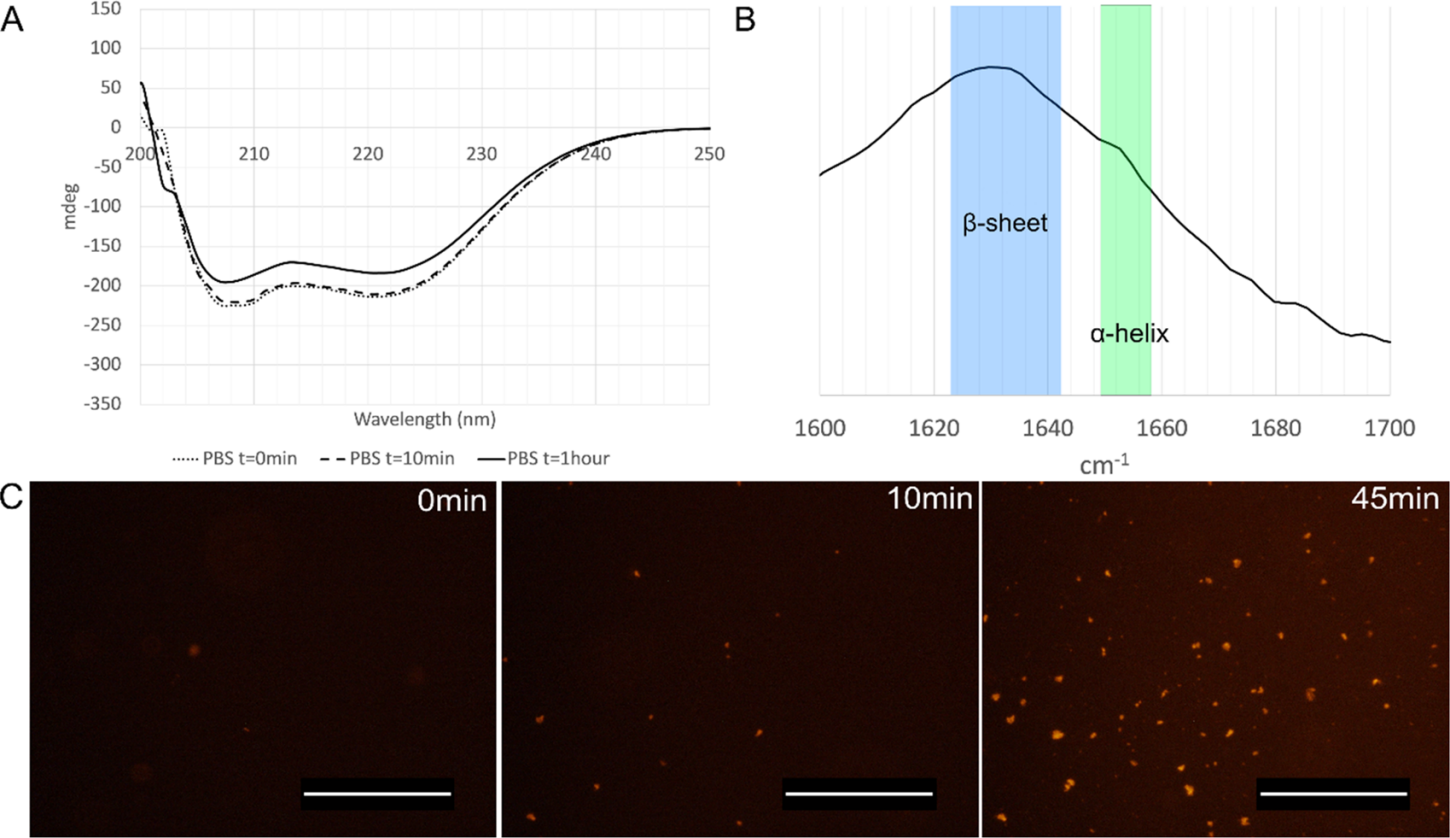
Secondary structure analysis of the FN-silk microspheres. (A) CD spectra of soluble FN-silk protein in PBS at *t* = 0 min, 10 min, and 1 h. (B) ATR-FTIR spectrum of FN-silk microspheres formed in PBS, isolated by centrifugation, and air-dried on a glass substrate. (C) Fluorescence microscopy images following the formation of FN-silk microspheres in PBS with Amytracker at time points 0, 10, and 45 min. Scale bar 200 μm.

Protein aggregation and assembly is often accompanied by changes in secondary structure. Three different techniques were used with the aim to monitor the structural transitions that occur during self-assembly of FN-silk protein into microspheres (Figure 5). Circular dichroism (CD) spectroscopy was used to investigate the secondary structure of the FN-silk protein in soluble form. FN-silk protein (1 mg/mL) in PBS showed a far-UV CD spectrum with two valleys, one at 208 and one at 222 nm (Figure 5A), indicative of an α-helix-dominated secondary structure, similar to what has previously been reported for 4RepCT.^32^ Within 1 h, the canonical helical signal from soluble FN-silk underwent absorption flattening, often referred to as Duyens flattening,^52,53^ which is related to anisotropic absorption due to particle formation. The observed change coincided with the observed loss of soluble protein and appearance of silk microspheres, which scatter the light, thereby decreasing the CD signal. Unfortunately, the HA molecule in solution gave a strong signal with a main valley at 209 nm in the far-UV region of the CD spectra (SI Figure S6, left), shielding possible changes of the FN-silk protein in this environment. The CD spectra of an HA gel with FN-silk added looked similar to HA gel alone but with a decrease in amplitude after a short period of time (10 min) and a shift of the negative peak, likely due to scattering from formed microspheres inside the hydrogel (SI Figure S6, right). This shift could be correlated to an underlying secondary structure transition toward a higher β-sheet content, as similar initial and final CD spectra have previously been reported for recombinant spider silk, representing such a transition from α-helical to β-sheet dominant content.^54^ Herein, CD spectroscopy could be used only to observe the loss of soluble helical protein. Other methods were needed to study the secondary structures of the formed microspheres.

Attenuated total reflectance Fourier transform infrared spectroscopy (ATR-FTIR) was used to analyze the secondary structure of the silk microspheres. FTIR spectra in the amide I region of dried layers of isolated microspheres formed in PBS showed a clear signal around 1623–1641 cm^–1^, commonly associated with the presence of β-sheet structures.^55^ β-Sheet structures are commonly found in amyloid fibrils, and their properties have previously been compared between recombinant major ampullate silk spidroin eADF16 and amyloid fibrils.^56^ A shoulder can be observed in the position of the α-helical fold (1648–1657 cm^–1^^57^) (Figure 5B). Also with this technique, the HA molecule gave a strong peak in the amide I region (SI Figure S7), which makes it difficult to discern the signal from the FN-silk in microspheres formed in HA-solution.

As a complementary method for investigation of protein structural changes, a commercially available luminescence conjugated pentameric formyl thiophene acetic acid (pFTAA), called Amytracker, was used. Amytracker has previously been shown to bind to protein aggregates with repetitive arrangements of β-sheets and fluoresce upon such binding.^58,59^ Herein, Amytracker was added to the protein solution to follow the assembly process of FN-silk into microspheres in PBS (Figure 5C). No fluorescence could be seen until the formation of the FN-silk microspheres, which agrees with the self-assembly observations made earlier with optical microscopy and the respective CD spectra. More specifically, a few microspheres could be seen within the first 10 min of incubation, with an increased amount after 45 min. The fluorescence thus confirmed the previous ATR-FTIR observation of a strong presence of β-sheets in the assembled spheres but in this case for wet conditions.

### Functionalized Silk Microspheres As Additives in 2D and 3D Cell Culture

**Figure 6:**
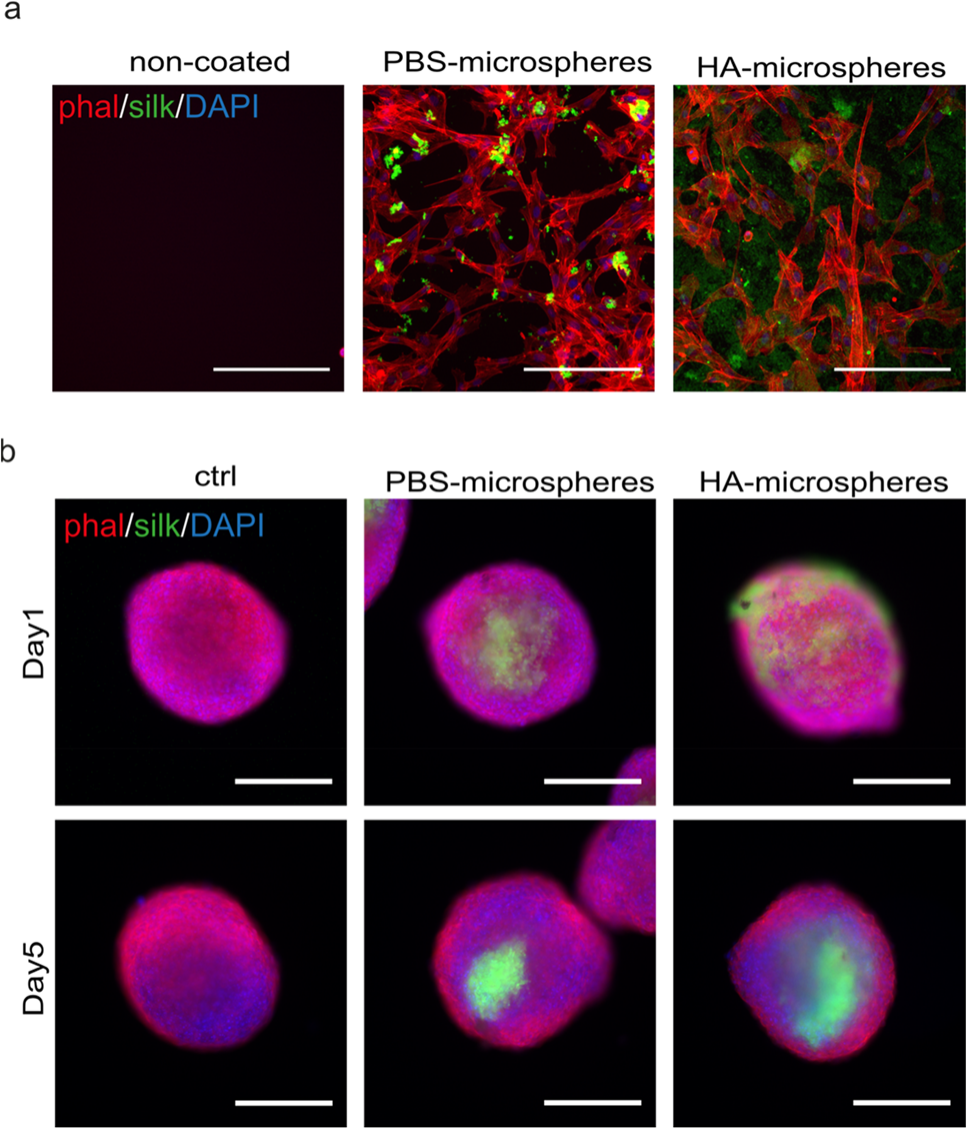
Integration of FN-silk microspheres in 2D and 3D cell cultures. (a) hMSCs attached and spread out on hydrophobic 2D surfaces with silk microspheres (green) formed in PBS (middle) or HA solution (right) but not without microspheres (left). F-actin staining of hMSCs with phalloidin (red) and nuclear counterstaining with DAPI (blue) at 24 h after seeding. Scale bar: 200 μm. (b) Integration of silk microspheres (green) during hMSC spheroid formation in a 96-well plate. Staining with Phalloidin (red) and DAPI (blue) at 1 day (upper panel) and 5 days (lower panel) after seeding. Scale bar 300 μm.

As the first example of an application, we examined the potential usage of silk microspheres as adhesion points for cells to be cultured on a hydrophobic 2D surface. FN-silk microspheres were produced, isolated as previously described, and dried onto the bottom of a hydrophobic 24-well plate before seeding human mesenchymal stem cells (hMSCs). After 1 day, the cells were fixated and stained for F-actin (phalloidin) and nuclei (DAPI) to visualize cell adhesion and spreading. In wells with silk microspheres, the hMSCs attached and spread normally over the surface. This accounted for cells grown in wells with microspheres formed either in PBS or in HA solution (Figure 6A). From these experiments, it was again apparent that the microspheres formed in PBS were larger and that both classes of spheres tended to cluster. The microspheres formed in HA solution displayed a more spread out and dim appearance on the surface (SI Figure S8A). This phenomenon could be due to the protrusions previously seen in TEM images (Figure 4, SI Figure S5) and could also involve potentially leftover HA molecules. As expected, no cells attached on the hydrophobic wells without silk microspheres (negative control), although the same number of cells was seeded. Thus, we conclude that the FN-silk microspheres promote cell attachment and can be used as an additive to biofunctionalize a hydrophobic surface.

Next, we investigated if FN-silk microspheres could be integrated into spheroids of cells in 96-well plates as a potentially scalable 3D cultivation format. For this, a conventional protocol for scaffold-free formation of spheroids from a single cell suspension in ultralow attachment (ULA) U-bottom wells with and without a fraction of FN-silk microspheres was used. The cell spheroids were cultured for up to 5 days to investigate the effect of microspheres on cell clustering, spheroid geometry, and growth (Figure 6b). No apparent difference in spheroid formation time, size, or shape could be observed. From the fluorescence signal of the labeled FN-silk (Figure 6b and SI Figure S8b), it could be concluded that the silk microspheres, from both PBS and HA solution, were efficiently integrated within the cell spheroids during formation. These spheroids stayed intact, with visible silk microspheres at their centers throughout the entire culture period. With this information, we could optimize cell culture conditions in the presence of FN-silk microspheres and gain a better understanding of the cellular responses to such an additive.

## Conclusions

Herein, we have used several advanced imaging and structural analysis methods to investigate the self-assembly behavior of FN-silk protein into microspheres in the bulk of PBS and HA-solution, with some observations also within HA cross-linked into a gel. We conclude that the presence of HA can be used to expedite the formation and control the size and size distribution of the self-assembled silk microspheres. The silk microspheres were found to be homogeneously filled, with some porosity. A substantial β-sheet content was observed, which is in line with previous observations of other FN-silk formats formed at solid/solid and solid/liquid interfaces. Some protrusions were found on the surface. Further investigation is required to examine the role that HA plays in this. Furthermore, we demonstrate the possible applications of FN-silk microspheres for biofunctionalization of surfaces to be used for *in vitro* cell culture or as implants. Finally, we show that silk microspheres can be efficiently integrated into cell spheroids, which opens avenues for biomedical applications such as targeted drug or growth factor delivery.

## Supporting Information Available

Additional microscopy images, CD-spectra, and ATR-FTIR spectra (PDF)
