# Supplementary material for: Self-Assembly of RGD-Functionalized Recombinant Spider Silk Protein into Microspheres in Physiological Buffer and in the Presence of Hyaluronic Acid
Source: ACS Appl Bio Mater. 2023 Aug 14;6(9):3696–705. doi: 10.1021/acsabm.3c00373 (PMC10521021; doi:10.1021/acsabm.3c00373)
Supplement: Supplementary file 1 — mt3c00373_si_001.pdf [file mt3c00373_si_001.pdf]

Supporting information

Self-assembly of RGD-functionalized recombinant spider silk protein into microspheres in physiological buffer and in the presence of hyaluronic acid

Eirini Ornithopoulou[1], Carolina Åstrand[1,2], Linnea Gustafsson[2,3], Thomas Crouzier[4] and My Hedhammar[1*]

[1] Department of of Protein Science, School of Chemistry, Biotechnology and Health (CBH), KTH Royal Institute of Technology, SE-106 91, Stockholm, Sweden

[2] Spiber Technologies AB, Roslagstullsbacken 15, 114 21 Stockholm, Sweden

[3] Division of micro and nanosystems, School of Electrical Engineering and Computer Science (EECS), KTH Royal Institute of Technology, SE-106 91, Stockholm, Sweden

[4] Department of Chemistry, School of Chemistry, Biotechnology and Health (CBH), KTH Royal Institute of Technology, SE-106 91, Stockholm, Sweden

[*] Corresponding author: myh@kth.se

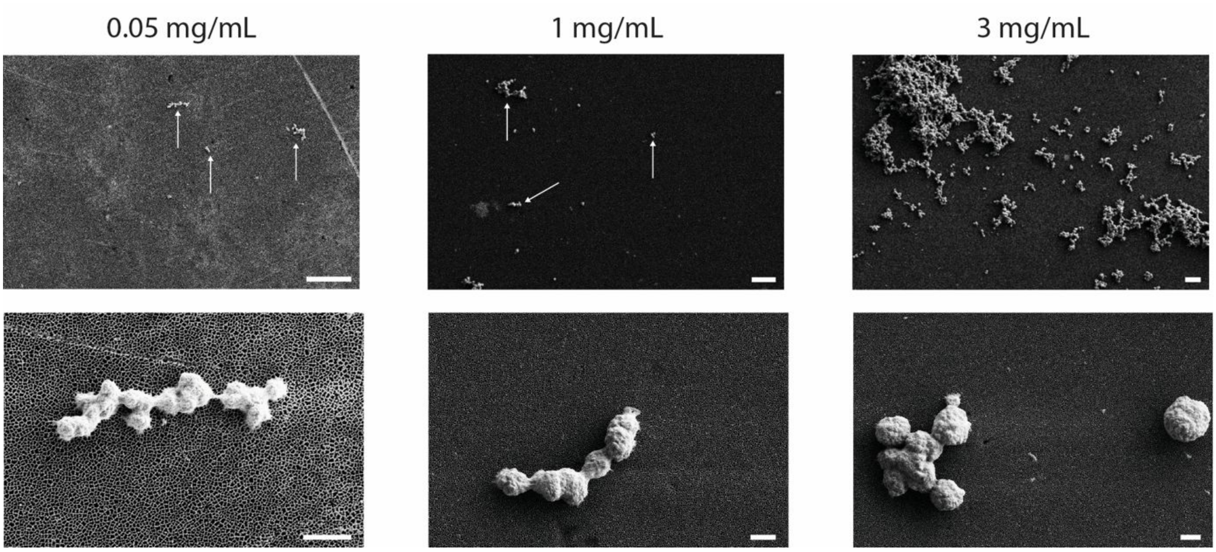

Figure S1. SEM images of microspheres formed at different concentrations of FN-silk in PBS. *Top row: Images taken of microspheres prepared using 0.05, 1 and 3 mg/ml where the white arrows point at clusters of microspheres, scale bar = 20 µm. Lower row: magnification of microspheres from top row, scale bar = 2 µm.*

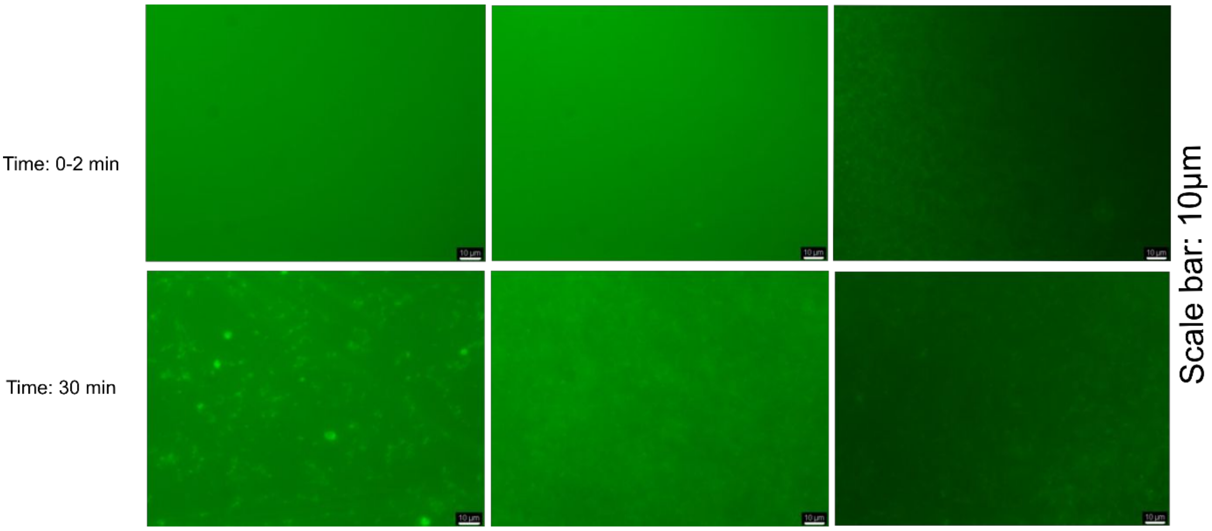

Figure S2. Fluorescence microscopy imaging of self-assembly of FN-silk protein into microspheres. *Top row: Images taken directly (0-2 min) after sample preparation of FN-silk (1 mg/ml) in 1xPBS buffer (PBS), hyaluronic acid (1.6%) dissolved in PBS (HA-solution) and HA cross-linked into a gel (HA-gel). Lower row: Images of the respective conditions after 30 minutes of incubation. Scale bar 10 µm.*

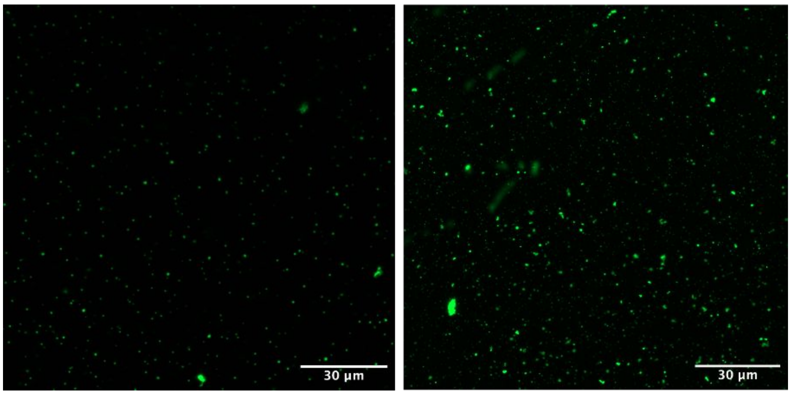

Figure S3. Confocal images of silk microspheres. *Silk microspheres (green) in HA gel after Day 1 (Left) and Day 6 (Right). Scale bar 30 µm.*

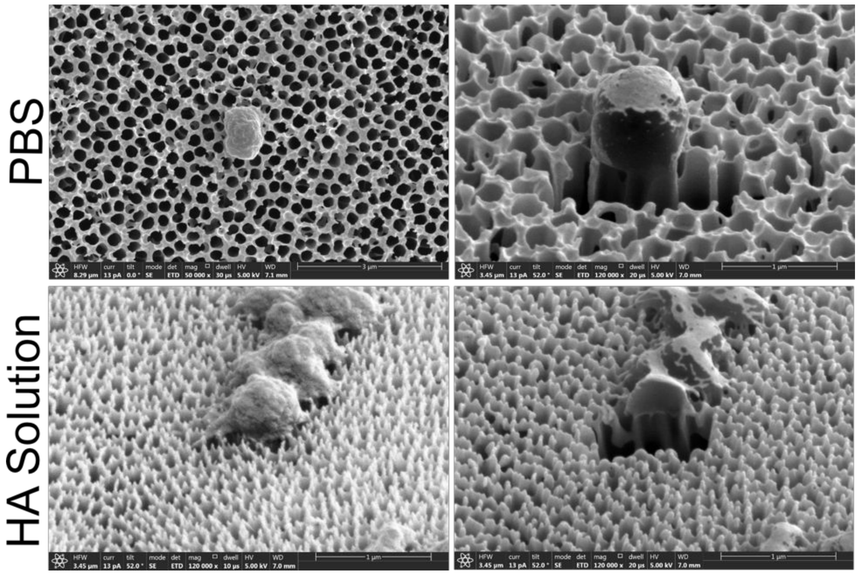

Figure S4. SEM images of silk microspheres formed in two different environments. *Top row: Microspheres from PBS. Bottom row: Microspheres from HA solution. Right column shows microspheres after milling with FIB.*

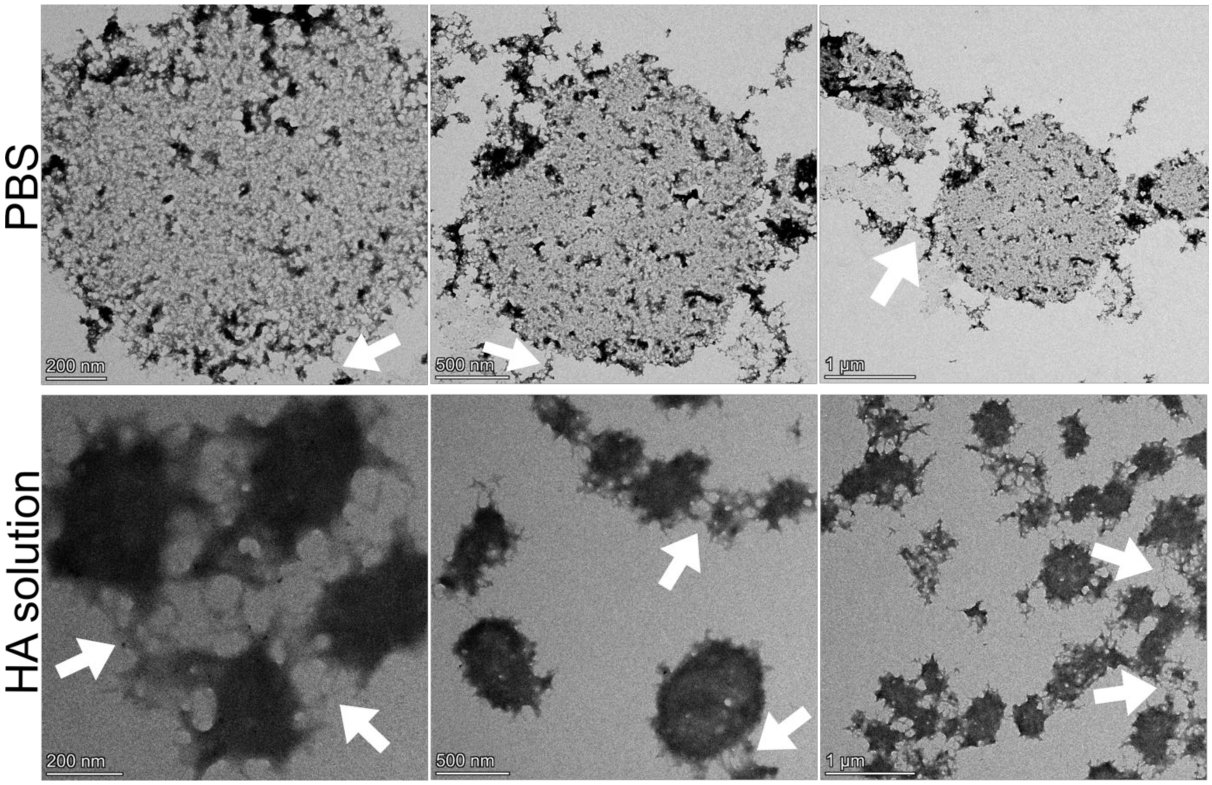

Figure S5. TEM images of cryo-sections of silk microspheres formed in two different environments. *Top row: Microspheres from PBS. Bottom row: Microspheres from HA solution.*

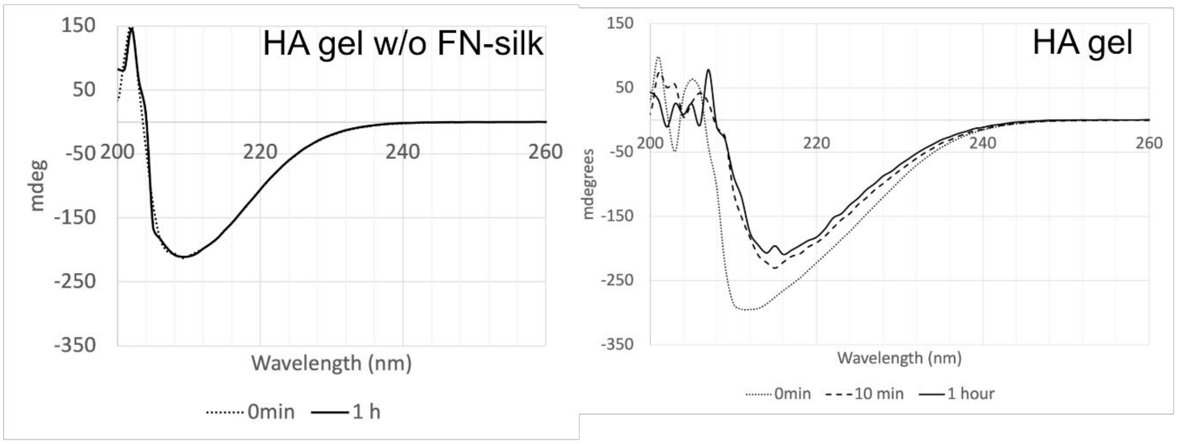

Figure S6. CD spectra of crosslinked HA with and without FN-silk, during the first hour of assembly. *Left: HA crosslinked hydrogel at 0 min and 1hour. Right: Crosslinked hydrogel at times: 0 min,10min and t=1 hour.*

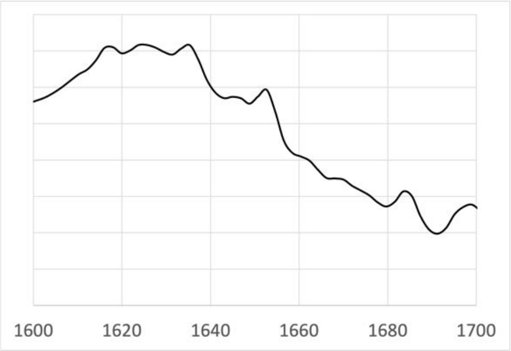

Figure S7. ATR-FTIR spectrum of HA solution without FN-silk.

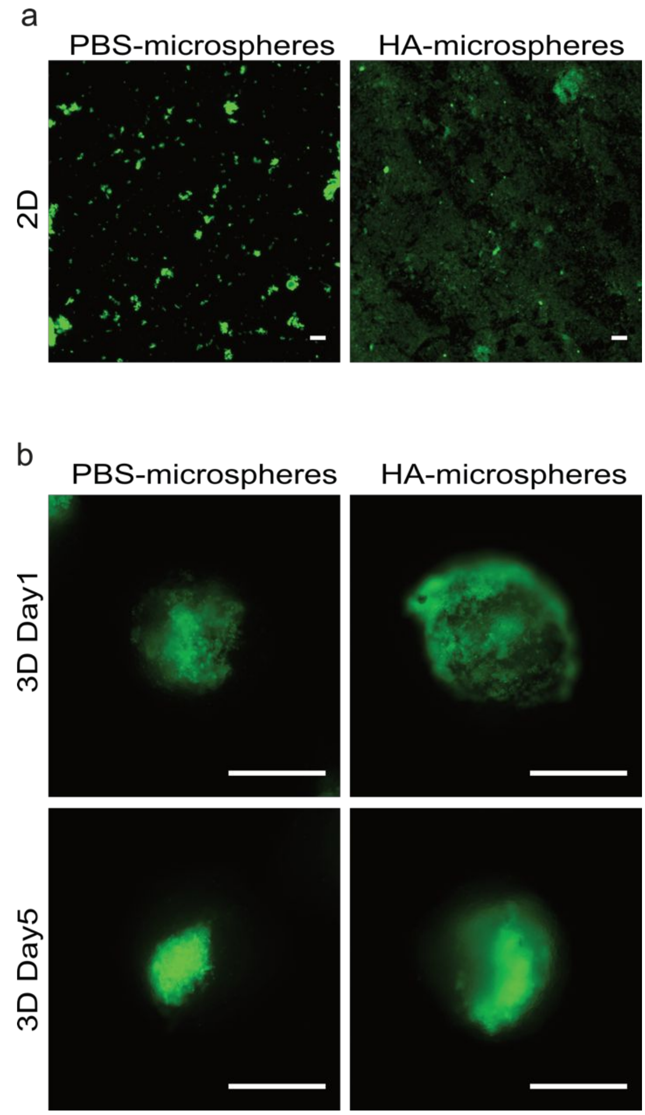

Figure S8. FN-silk microspheres in 2D and 3D cell culture experiments. *(a) Images of 488-labeled silk microspheres formed in PBS (left) or in HA (right) attached onto hydrophobic surfaces at 24h after cell seeding. Scale bar 20 µm. (b) Integration of 488-labeled silk microspheres (green) into hMSC spheroids formed in a U-shaped 96-well at day 1 (upper panel) and day 5 (lower panel). Scale bar 300 µm.*
